# Mild Anemia May Affect Thyroid Function in Pregnant Chinese Women During the First Trimester

**DOI:** 10.3389/fendo.2021.772917

**Published:** 2021-12-09

**Authors:** Guan-ying Nie, Rui Wang, Peng Liu, Ming Li, Dian-jun Sun

**Affiliations:** ^1^ Key Lab of Etiology and Epidemiology, National Health Commission & Education Bureau of Heilongjiang Province (23618504), Key Laboratory of Trace Elements and Human Health, Center for Endemic Disease Control, Chinese Center for Disease Control and Prevention, Harbin Medical University, Harbin, China; ^2^ Examination Department, Central Hospital Affiliated to Shenyang Medical College, Shenyang, China

**Keywords:** the first trimester, mild anemia, FT4, TSH, reference values, subclinical hypothyroidism

## Abstract

**Background:**

Pregnant women are often susceptible to anemia, which can damage the thyroid gland. However, compared with moderate and severe anemia, less attention has been paid to mild anemia. The purpose of this study was to evaluate the effect of mild anemia on the thyroid function in pregnant women during the first trimester.

**Methods:**

A total of 1,761 women in the first trimester of their pregnancy were enrolled from Shenyang, China, and divided into mild anemia and normal control groups based on their hemoglobin levels. Thyroid-stimulating hormone (TSH), free thyroxine (FT4), and free triiodothyronine (FT3) levels were compared between the two groups.

**Results:**

The TSH levels of pregnant women with mild anemia were higher than those of pregnant women without mild anemia (p < 0.05). Normal control women were selected to set new reference intervals for TSH, FT3, and FT4 levels during the first trimester, which were 0.11–4.13 mIU/l, 3.45–5.47 pmol/l, and 7.96–16.54 pmol/l, respectively. The upper limit of TSH 4.13 mU/l is close to the upper limit 4.0 mU/l recommended in the 2017 American Thyroid Association (ATA) guidelines, indicating that exclusion of mild anemia may reduce the difference in reference values from different regions. Mild anemia was related to 4.40 times odds of abnormally TSH levels (95% CI: 2.84, 6.76) and 5.87 increased odds of abnormal FT3 (95% CI: 3.89, 8.85). The proportion of hypothyroidism and subclinical hypothyroidism in patients with mild anemia was higher than that in those without anemia (0.6% vs. 0, p = 0.009; 12.1% vs. 1.9%, p < 0.001). Mild anemia was related to 7.61 times increased odds of subclinical hypothyroidism (95% CI: 4.53, 12.90).

**Conclusions:**

Mild anemia may affect thyroid function during the first trimester, which highlights the importance of excluding mild anemia confounding when establishing a locally derived specific reference interval for early pregnancy.

## Introduction

Thyroid dysfunction during pregnancy increases the risk of miscarriage, premature birth, fetal malformation, and even fetal death (1, 2). Therefore, to maintain maternal and child health, thyroid function should be actively advocated for all pregnant women.

Anemia is defined as a hemoglobin level of 120 g/l or less in women, and 110 g/l or less in pregnant women (3–5). The World Health Organization (WHO) has reported that 23% of pregnant women in industrialized countries and 52% in non-industrialized countries suffer from anemia (6). Iron deficiency (ID) is the main cause of anemia (7). Other causes include lack of vitamin A, B12, or folic acid; infectious diseases such as malaria and AIDS; and other hereditary anemias such as sickle cell disease (8). Anemia and thyroid function studies carried out in animal models have verified that low iron intake significantly decreases hemoglobin (Hb) levels, thyroid peroxidase (TPO) activity (9, 10), and serum concentrations of T3 and T4 (11), while increasing TSH levels (12). In 2007, Zimmermann et al. first reported that ID was associated with higher TSH and lower T4 levels in Swiss pregnant women (13). In recent years, studies in China have shown that ID can also be associated with abnormal thyroid function of pregnant women (14–17). Additionally, hypothyroidism occurs in patients with chronic hemolytic anemia, and its incidence is positively correlated with age and the severity of anemia (18).

Nevertheless, insufficient attention has been directed toward the relationship between mild anemia and thyroid function in pregnant women. Mild, moderate, and severe anemia in women was defined as a hemoglobin level of 110–119, 80–109, and <80 g/l, while that in pregnant women was defined as 100–109, 70–99, and <70 g/l (3–5). A study has revealed that pregnant women with mild, moderate, or severe anemia are related to infant anemia (19). Compared to those of moderate and severe anemia, the symptoms of mild anemia are non-specific and are only clinically detectable, which might explain why little attention is paid to mild anemia. A study has reported the prevalence of anemia among Chinese women before pregnancy to be 21. 64% (mild: 14.10%, moderate: 7.17%, and severe: 0.37%) in 2017 (20). According to the results monitored from 2010 to 2012, the anemia rate in pregnant women in China was 17.2%, while mild anemia accounted for approximately 61.0% (21). Mild anemia accounted for the highest proportion of overall anemia. Therefore, this study aimed to explore the effect of mild anemia in early pregnancy on the thyroid function of pregnant women in Shenyang, a city in northeast of China.

## Materials and Methods

### Study Population

**Figure 1:**
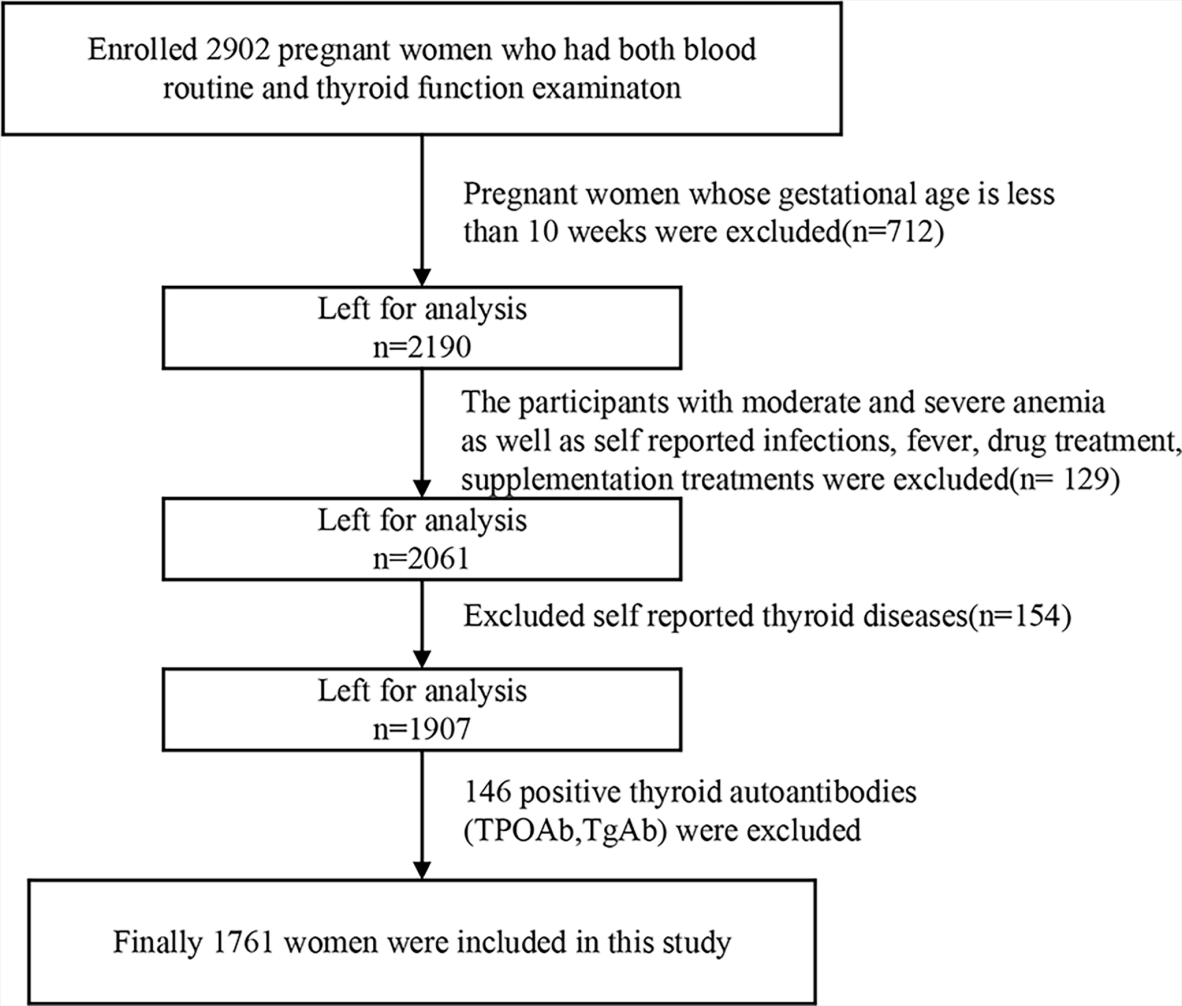
Flowchart of participants screening process.

The study was approved by the Ethics Committee of Harbin Medical University. We extracted the first routine evaluation results of pregnant women in the obstetric outpatient department of the Affiliated Central Hospital of Shenyang Medical College from September 2011 to December 2018. Prior to enrollment into the study, the pregnant women underwent routine blood and thyroid function examination (n = 2,902). Women with a pregnancy of 10–12 weeks of gestation were included in the study. Those women with a pregnancy of less than 10 weeks or more than 12 weeks of gestation were not eligible to participate (n = 712). Pregnant women with moderate anemia, severe anemia, self-reported blood diseases, infections, fever, drug treatment, or supplement treatment (n = 129) were also excluded. Pregnant women with self-reported thyroid disease (n = 154) or positive thyroid autoantibodies (TPOAb, TgAb, or TMAb) (n = 146) were also considered to be ineligible. Based on the abovementioned criteria, a total of 1,761 pregnant women were selected, including 314 pregnant women with mild anemia and 1,447 pregnant women without anemia as the control group (**Figure 1**).

### Method

Venous blood samples were collected in the morning after overnight fasting for more than 8 h. Blood samples were centrifuged to obtain serum, which was sent to the clinical laboratory for testing. The levels TSH, FT4, and FT3 were determined in all subjects on the same day of sampling, using a commercial electrochemiluminescence immunoassay kit (Beckman UniCel DxI800 automatic chemiluminescence analyzer, USA). Hemoglobin (Hb) levels were measured using an automated hematology analyzer XE-2100 (Sysmex Diagnostics, Japan). The women were considered to have mild anemic if their Hb was determined to be in the range 100–109 g/l.

### Statistical Analysis

Statistical analysis was performed using the Statistical Product and Service Solutions (SPSS) software (version 17.0, Chicago, IL). The Kolmogorov–Smirnov test was performed to confirm normality. Normally distributed FT4 were represented as mean ± standard deviation (SD), while FT3 and TSH did not follow a Gaussian distribution which were represented by a median (minimum–maximum). For normally distributed data, a t-test was applied to compare groups. For non-normal data, a Kruskal–Wallis test and subsequent Mann–Whitney test were used to compare groups. When comparing the rates between groups, we use the chi-square test. We used univariable and multivariate logistic regression to examine the significant influence factors for thyroid function. A two-tailed p-value < 0.05 was considered statistically significant. Statistical power analyses were done using G*Power 3.1 (22). According to NCCLS C28-A2, the 2.5th and 97.5th percentiles were considered as the lower and upper limits, respectively, of the reference intervals (23). The 95% confidence interval (CI) of the two limits was obtained through bootstrap by R 4.0.2.

## Results

### Subject Characterization

In total, 1,761 individuals with a mean age of 29.37 ± 3.84 years participated in this study. The average gestation age was 11 ± 1 week. The median TSH level was 1.6 mIU/l (range 0.01, 9.67 mIU/l), the median FT4 level was 12.57 pmol/l (range 1.15, 23.06 pmol/l), and the median FT3 level was 4.26 pmol/l (range 2.67, 13.15 pmol/l). The prevalence of mild anemia among all women was 17.8% (314/1761).

### Different Distribution of Thyroid Hormones During the First Trimester Between Women With Mild Anemic and Control Pregnant Women

We compared the thyroid function values between pregnant women with mild anemia and the control group and found that there was no difference in the levels of FT3 and FT4 between the two groups. The TSH levels in pregnant women with mild anemia were higher than those in pregnant women without anemia (p < 0.001), as shown in **Table 1**.

**Table 1:** Comparison of thyroid functions between pregnant women with mild anemia and control group.

Thyroid function	Pregnant women		*p value*
	Control (n = 1447)	Mild anemia (n = 314)	
FT3 (pmol/L), median [min, max]	4.24 [2.67, 5.94]	4.35 [3.11, 13.15]	**0.05*^a^***
FT4 (pmol/L), mean ± SD	12.43 ± 2.29	12.49 ± 2.34	**0.655*^b^***
TSH (mIU/L), median [min, max]	1.58 [0.01, 6.47]	1.72 [0.01, 9.67]	<0.001^a^*

^a^Mann–Whitney U-test was used to compare the groups.

^b^T-test was used to compare the groups.

^*^Statistical significance was assumed when the p-value was < 0.05.

### Reference Range for Thyroid Hormones During the First Trimester in Pregnant Women

We choose pregnant women in the control group to set a new reference interval. As shown in **Table 2**, the TSH ranges from 0.11 to 4.13 mIU/l, FT3 from 3.45 to 5.47 pmol/l, and FT4 from 7.96 to 16.54 pmol/l. The standard reference ranges provided by manufacturers are as follows: TSH ranges from 0.68 to 5.59 mIU/l, FT3 from 3.28 to 6.47 pmol/l, and FT4 from 7.64 to 16.03 pmol/l. We selected pregnant women from both the control group and the group comprising women with mild anemia to calculate a reference interval, wherein the range of TSH was considered to be from 0.13 to 4.65 mIU/l, FT3 from 3.43 to 5.91 pmol/l, and FT4 from 7.98 to 16.54 pmol/l.

**Table 2:** Reference ranges of thyroid function.

Thyroid function	n	P2.5	95% CI (P 2.5)	P 97.5	95% CI (P 97.5)
FT3 (pmol/L)					
Standard		3.28		6.47	
Pregnant women	1761	3.43	3.39–3.46	5.91	5.81–6.36
New	1447	3.45	3.41–3.49	5.47	5.42–5.56
FT4 (pmol/L)					
Standard		7.64		16.03	
Pregnant women	1761	7.98	7.85–8.16	16.54	16.27–16.90
New	1447	7.96	7.79–8.09	16.54	16.28–16.86
TSH (mIU/L)					
Standard		0.68		5.59	
Pregnant women	1761	0.13	0.06–0.44	4.65	4.56–5.03
New	1447	0.11	0.07–0.18	4.13	3.98–4.26

Standard, the reference ranges provided by manufacturers.

Pregnant women, pregnant women from both the control group as well as the group comprising women with mild anemia were selected to calculate a reference interval.

New, only chose control pregnant women to set the new reference interval.

On comparing our new reference with the standard references, serum TSH levels were decreased in the first trimester, with the upper limit declining by 26.2% and the lower limit declining by 83.4%. The upper limit of serum FT3 declined by 15.5%, and the lower limit increased by 5.2%. Serum FT4 showed an increase too, with the upper and lower limits raised by 3.2% and 4.2%, respectively.

Comparison between the new reference with the references including mild anemia demonstrated the following changes. Serum TSH levels decreased in the first trimester, with the upper limit declining by 11.1% and the lower limit declining by 15.4%, while the serum FT3 upper limit declined by 7.5%.

### TSH and FT3 but Not FT4 May Be Affected by Mild Anemia in Early Pregnancy

Based on the reference range of healthy control pregnant women, we compared the indicators of thyroid function between pregnant women with mild anemia and the control group. As shown in **Table 3**, the rate of TSH values above the upper limit in pregnant women with mild anemia was notably higher than that in the control (12.7% vs. 1.9%, *p* < 0.0001). The percent of FT3 values under the lower limit or above the upper limit in pregnant women with mild anemia was greater than that in the control (3.8% vs. 1. 9%, 15.0% vs. 1.9%, *p* < 0.0001). In addition, there was no significant difference between the FT4 values.

**Table 3:** Comparison of thyroid functions abnormal between pregnant women with mild anemia and control group.

	Pregnant women		Chi-square χ2	*p value*
	Control (n = 1447)	Mild anemia (n = 314)		
	n (%)	n (%)		
FT3			84.240	<0.0001^*^
Low	27 (1.9%)	12 (3.8%)		
Normal	1392 (96.2%)	255 (81.2%)		
High	28 (1.9%)	47 (15.0%)		
FT4			0.617	0.734
Low	28 (1.9%)	5 (1.6%)		
Normal	1391 (96.1%)	301 (95.9%)		
High	28 (1.9%)	8 (2.5%)		
TSH			60.064	<0.0001^*^
Low	28 (1.9%)	6 (1.9%)		
Normal	1391 (96.1%)	268 (85.4%)		
High	28 (1.9%)	40 (12.7%)		

^*^Statistical significance was assumed when the p-value was < 0.05.

As shown in **Table 5**, mild anemia [OR 4.26 (95% CI: 2.82, 6.43); p < 0.001)] was independently associated with a higher risk for abnormal TSH levels. After adjustment for confounding variables in the final step forward logistic regression, mild anemia (OR 4.40 (95% CI: 2.84, 6.76); p < 0.001) was independently associated with a higher risk for abnormal TSH levels. Mild anemia (OR 5.86 (95% CI: 3.96, 8.67); *p* < 0.001) was independently associated with a higher risk for FT3 abnormality risk. In addition, age >35 years (OR 2.45 (95% CI: 1.30, 4.33); *p* < 0.001) was associated with a higher FT3 abnormality risk. After adjustment for confounding variables in the final step forward logistic regression, mild anemia (OR 5.87 (95% CI: 3.89, 8.85); *p* < 0.001) was independently associated with a higher FT3 abnormality risk. Age >35 years was no longer associated with a higher risk for FT3 abnormal.

### Mild Anemia May Be Related to Subclinical Hypothyroidism in Early Pregnancy

As shown in **Table 4**, the prevalence of subclinical hypothyroidism was markedly higher in pregnant women with mild anemia than in the control group (12.1% vs. 1.9%, *p* < 0.001). Moreover, there were two (0.64%) overt hypothyroidism cases in pregnant women with mild anemia, but none was found in the control group. These differences were significant (*p* = 0.009). In contrast, there was no significant difference in the prevalence of subclinical hyperthyroidism and hyperthyroidism between the pregnant women with mild anemia and the controls. As shown in **Table 5**, mild anemia (OR 6.98 (95% CI: 4.23, 11.65); p < 0.001)) was independently associated with a higher risk for subclinical hypothyroidism. After adjustment for confounding variables in the final step forward logistic regression, mild anemia (OR 7.61 (95% CI: 4.53, 12.90); p < 0.001) was independently associated with a higher risk for subclinical hypothyroidism.

**Table 4:** Prevalence of thyroid diseases in pregnant women with mild anemia compared to the control group.

	Pregnant women		Chi-square χ2	*p value*
	Control (n = 1447)	Mild anemia (n = 314)		
	n (%)	n (%)		
Overt hypothyroidism, n (%)			6.907	0.009*
Yes	0 (0.0)	2 (0.6)		
No	1447 (100.0)	312 (99.4)		
Subclinical hypothyroidism, n (%)			54.903	<0.0001^*^
Yes	28 (1.9)	38 (12.1)		
No	1419 (98.1)	276 (87.9)		
Overt hyperthyroidism, n (%)			0.416	**0.519**
Yes	2 (0.1)	1 (0.3)		
No	1445 (99.9)	313 (99.7)		
Subclinical hyperthyroidism, n (%)			0.064	**0.800**
Yes	26 (1.8)	5 (1.6)		
No	1421 (98.2)	309 (98.4)		

^*^Statistical significance was assumed when the p-value was < 0.05.

**Table 5:** Results from the univariable and multivariable logistic regression analysis.

Dependent/independent	Univariable analysis		Multivariable analysis	
variables	Rough OR (95% CI)	*p value*	Adjust OR*^a^* (95% CI)	*p value*
Outcome: abnormal FT3 levels (logistic regression)				
Mild anemia	5.86 (3.96,8.67)	<0.0001^*^	5.87 (3.89,8.85)	<0.0001^*^
Age group (>35)	2.45 (1.30,4.33)	<0.0001^*^		
Outcome: abnormal FT4 levels (logistic regression)				
Age group (>35)	0.72 (0.17,1.99)	**0.589**		
Mild anemia	1.07 (0.55,1.92)	**0.823**		
Outcome: abnormal TSH levels (logistic regression)				
Mild anemia	4.26 (2.82,6.43)	<0.0001^*^	4.40 (2.84,6.76)	<0.0001^*^
Age group (>35)	1.83 (0.87,3.47)	**0.08**		
Outcome: subclinical hypothyroidism (logistic regression)				
Mild anemia	6.98 (4.23,11.65)	<0.0001^*^	7.61 (4.53,12.90)	<0.0001^*^
Age group (>35)	1.65 (0.62,3.62)	**0.25**		

^*^Statistical significance was assumed when the p-value was < 0.05.

^a^Adjust for age.

## Discussion

Anemia is a common disease that may occur during pregnancy. A meta-analysis study from China demonstrated that the prevalence of anemia in pregnant women during the first trimester was found to be 10.1% (95% CI 6.2%–14%) during the period from 2012 to 2016 (24). The monitoring results from 2011 to 2012 showed that the anemia rate of pregnant women in small and medium-sized Chinese cities is 18.0% (25). A study has also proven that the risk of anemia in pregnant women in the north is higher than that in the south region of China (21). Mild anemia accounted for the highest proportion of overall anemia. Our study comprised data from women in early pregnancy from February 2012 to December 2018 in Tiexi District of Shenyang and found a mild anemia rate of 17.8% in pregnant women during their first trimester. Our study found that the high incidence of mild anemia was closely related to higher maternal age (older than 35 years) [OR 10.88 (95% CI: 7.02, 17.12)] (data not shown). This finding is similar to that reported by Lin et al. Their study surveyed 43,403 pregnant women in Beijing, Chengdu, and Guangzhou and found that maternal anemia was significantly related to maternal age 35 years and older (AOR = 1.386) (26). Pregnancies at an advanced reproductive age are common now. Therefore, it is essential to give additional importance to the influence of mild anemia as well as anemia on health of pregnant women and fetus.

Fifty percent of overall anemia cases in populations is caused by ID (8). ID is frequent during the first trimester of pregnancy and is often related to a higher prevalence of thyroid autoimmunity, increased TSH, and lower FT3 levels (27). A study in Wuxi also confirmed that low iron stores showed a trend toward higher TSH, lower FT3, and lower FT4 levels during the first trimester of pregnancy (15). A study in Suzhou revealed that pregnant women with mild ID and ID anemia have higher TSH and lower FT4 status (16). These studies on TSH and FT3 are consistent with our study. In our study, we found that TSH levels were higher in pregnant women with mild anemia than in those without anemia (p < 0.001). According to our new intervals, we observed that mild anemia was independently associated with a higher risk for abnormal TSH and FT3 levels. In this study, we also found that the prevalence of subclinical hypothyroidism and overt hypothyroidism was markedly higher in women with mild anemia than in those without.

However, no differences were observed in FT4 values between pregnant women with mild anemia and those without it. The first reason may be that we excluded pregnant women with self-reported thyroid disease or positive thyroid autoantibodies during sample screening. These patients are the main contributors to the abnormal FT4 levels, which was standard for diagnosis of the thyroid disease. The second reason may be that previous studies focus on ID which includes not only mild anemia but also moderate and severe anemia. However, we just focus on mild anemia. Thirdly, lower FT4 levels may also be related to other factors such as iodine nutrition, age, and gestational weeks, which need to be further elucidated. Ipek et al. found no difference in FT4 levels between ID anemia children and normal children (28). Tienboon and Unachak found that there was no difference in T4, T3, fT4, fT3, thyroxine-binding globulin (TBG), and TSH levels before and after iron treatment in ID anemia children (29). Ravanbod et al. reported the absence of significant differences in Hb and TSH levels before and after 90 days of iron treatment in non-pregnant patients with ID anemia and subclinical hypothyroidism (SCH), which suggested that iron alone does not change the TSH level in non-pregnant patients with ID anemia and SCH (30). Infusion of concentrated red blood cells can increase Hb, thyroxine (T3), and free-T3 (FT3) levels in patients with thalassemia who possessed normal thyroid function before puberty, but it has no effect on patients with delayed puberty (31). Therefore, mild anemia might not be the only determining factor affecting thyroid function.

In our study, the temporality of the association between anemia and abnormal thyroid function could not be assessed because exposure and outcome were measured simultaneously. Nevertheless, anemia and thyroid function animal studies have demonstrated that low-iron food significantly decreased the Hb levels and TPO activity (9, 10), as well as serum concentrations of T3 and T4 (11), while a rise in TSH levels was observed (12). ID anemia resulted in maternal hypothyroxinemia from midgestation to the end of the pregnancy in pregnant rats (32). Interestingly, in Nepalese children, ID was also found to decrease the activity of TPO, an iron-containing enzyme involved in the synthesis of thyroid hormones (33). Iron therapy studies have implied that in ID adolescent girls, improvement in iron status was accompanied by an improvement in some indices of thyroid hormones (34). After treatment with iron, FT4 levels significantly increased in patients with ID anemia (35). Beard et al. reported that in women with ID anemia, iron supplementation corrected the anemia significantly (p = 0.03) improved the rectal temperature, and partially normalized the plasma thyroid hormone concentrations (36). Studies have emphasized that adding iron to thyroxine therapy improves both conditions compared to thyroxine therapy alone (30, 37). In addition, patients with chronic hemolytic anemia requiring repeated blood transfusion have a high prevalence of the hypothalamic–pituitary thyroid axis. Proper blood transfusion appears to prevent deterioration of thyroid function and, in many cases, can reverse thyroid pathology (18). Therefore, the significant auxiliary effect of mild anemia on thyroid function should not be ignored.

For the evaluation of thyroid function in pregnant women, there have been several studies and guidelines indicating that non-pregnant reference intervals for thyroid hormones are not applicable for pregnancy. There are many challenges in reference value formulation for thyroid function in pregnant women. Maternal human chorionic gonadotrophin (hCG) directly stimulates the TSH receptor, increasing thyroid hormone production by nearly 50%, resulting in a subsequent reduction in serum TSH concentration (38). The levels of thyroid hormones vary according to the gestational age (39). Moreover, the pregnancy reference intervals could be affected by race, kits, and test methods (40–42). It has been reported that the formulation of gestational reference ranges is always given to be inconsistent. The 2011 ATA guidelines suggested a specific upper limit cutoff (2.5 mU/l) for serum TSH levels in the first trimester of pregnancy (43). Nonetheless, the TSH upper limits given by studies worldwide are higher than 2.5 mU/l. Then, the 2017 ATA guidelines suggested an upper reference limit of 4.0 mU/l (1). Nevertheless, a series of studies have demonstrated that compared to the European and American population, or the reference value suggested by ATA, the Chinese pregnant population has a higher upper TSH limit (43–45).

As described above, our study implied that pregnant women with mild anemia had abnormally high TSH levels. According to ATA recommendation, making thyroid function reference ranges should only include pregnant women with no known thyroid disease, optimal iodine intake, and negative thyroid peroxidase antibody (TPOAb) status (1). Conversely, they did not consider excluding pregnant women with mild anemia by clinically Hb detection. In our study, we excluded mild anemia and established a new first trimester-specific reference interval for pregnant women. Interestingly, the upper limit of 4.13 mU/l obtained was almost equal to the upper limit of 4.0 mU/l given by the 2017 ATA guideline (1). Therefore, inconsistent reference ranges worldwide may not only be due to race and iodine nutrition but also be due to the diverse degrees of mild anemia rate among different countries. Pregnant women with mild anemia are easily overlooked due to their symptoms which are non-specific and obscure. In addition, pregnant women with mild anemia during the first trimester may be healthy before pregnancy, and some symptoms of mild anemia are similar to general pregnancy symptoms. Therefore, mild anemia cannot be ruled out without routine blood examinations.

After excluding mild anemia, the upper and lower limits of serum TSH decreased. Therefore, if the reference interval is calculated with anemia confounding, the diagnosis of high TSH levels related to hypothyroidism in pregnant women will be missed, and the diagnosis of lower TSH levels related to hyperthyroidism in pregnant women will be misdiagnosed. After excluding mild anemia, the serum FT3 upper reference limit declined by 7.5%. Thus, if reference values were calculated with mild anemia included, the diagnosis of high FT3 levels related to thyroid function in pregnant women would be missed.

In addition, according to the 2017 ATA guidelines, the upper TSH limit of 4.0 mU/l represents a reduction of approximately 0.5 mU/l from the non-pregnant upper limit (1). The new reference value of 4.13 mU/l from our study represents a relatively decent rate of reduction (26%) from the non-pregnant TSH upper reference limit (5.59 mIU/l) rather than a reduction of 0.5 mU/l. A systematic review by Gao et al. also emphasized that pregnant women had a 22% reduction in the serum TSH upper limit from the non-pregnant value (42). This can be used as a suboptimal method to determine the threshold value for pregnant women in the first trimester.

It should be mentioned that the present study has several limitations, which need to be improved in further studies. First, certain parameters such as serum ferritin (SF), soluble transferrin receptor (sTfR), and total body iron (TBI) were not measured; the mild anemia status assessment would be more accurate with the measurement of these parameters. Secondly, this hospital-based study prevented us from obtaining detailed information on lifestyle factors and dietary habits, which may have a critical effect on the causal relationship between maternal thyroid function and mild anemia. Thirdly, pregnant women analyzed in the present study were not randomly selected, and the sample sizes of control and mild anemia groups were not equal, possibly introducing a selection bias. Although there is sufficient statistical power to investigate the association between abnormal thyroid function risk and anemia (see in **Supplementary Table 1**), larger prospective and different phases of pregnancy trials based on multicenters or community to replicate these findings are needed in the future. Fourth, the cross-sectional design of measuring exposure and results at the same time is the main limitation of this study; hence, it is impossible to assess the timeliness of the association between anemia and abnormal thyroid function. Therefore, in order to better understand the impact of mild anemia on thyroid function, the molecular mechanism should be studied based on animal models with different degrees of anemia during pregnancy and cell experiments in the future.

In conclusion, this study suggested a possible association between mild anemia and abnormal thyroid function in pregnant women during the first trimester; therefore, physicians should be aware of mild anemia during the first trimester to avoid adverse pregnancy outcomes. Moreover, the interval made by pregnant women without mild anemia is closer to the 2017 ATA reference, which indicated that the difference in TSH value in pregnant women globally might be partly due to the different incidence rates of mild anemia in pregnant women around the world. Therefore, formulation of thyroid hormones reference for pregnant women should exclude those with mild anemia, which need further study.

## Data Availability Statement

The original contributions presented in the study are included in the article/**Supplementary Material**. Further inquiries can be directed to the corresponding authors.

## Ethics Statement

The studies involving human participants were reviewed and approved by the Ethics Committee of Harbin Medical University. The patients/participants provided their written informed consent to participate in this study.

## Author Contributions

ML and D-jS were involved in conception and design of the research. ML drafted the manuscript. G-yN and RW performed the experiments and analyzed the data. G-yN and ML prepared the figures and tables. ML interpreted the results of the experiments. G-yN, RW, PL, ML, and D-jS edited and revised the manuscript and approved the final version of the manuscript.

## Funding

This study was supported by the Heilongjiang Natural Science Foundation (Project TD2019H001) and Harbin Medical University Funding (Project HMUMIF21015).

## Conflict of Interest

The authors declare that the research was conducted in the absence of any commercial or financial relationships that could be construed as a potential conflict of interest.

## Publisher’s Note

All claims expressed in this article are solely those of the authors and do not necessarily represent those of their affiliated organizations, or those of the publisher, the editors and the reviewers. Any product that may be evaluated in this article, or claim that may be made by its manufacturer, is not guaranteed or endorsed by the publisher.

## Supplementary Material

Supplementary Table 1 (total sample size = 1761, two-side alpha = 0.05). ^a^ Allocation ratio equal to 14.4474, 16.2647 and 25.68 for FT3, TSH and Subclinical hypothyroidism, respectively.
