# Supplementary material for: Mild Anemia May Affect Thyroid Function in Pregnant Chinese Women During the First Trimester
Source: Front Endocrinol (Lausanne). 2021 Dec 9;12:772917. doi: 10.3389/fendo.2021.772917 (PMC8695550; doi:10.3389/fendo.2021.772917)
Supplement: Supplementary Table 1 — (total sample size = 1761, two-side alpha = 0.05). a Allocation ratio equal to 14.4474, 16.2647 and 25.68 for FT3, TSH and Subclinical hypothyroidism, respectively. [file Table_1.docx]

Supplement table 1 (total sample size = 1761, two-side alpha = 0.05)

| ORs |  | Power ^a^ | |
| --- | --- | --- | --- |
|  | FT3 abnormal | TSH abnormal | Subclinical hypothyroidism |
| 2 | 0.8409313 | 0.819818 | 0.6658356 |
| 3 | 0.9979434 | 0.996727 | 0.9715405 |
| 4 | 0.9999899 | 0.999975 | 0.9986312 |
| 5 | 1 | 0.9999998 | 0.9999435 |

^a^ Allocation ratio equal to 14.4474, 16.2647 and 25.68 for FT3, TSH and Subclinical hypothyroidism, respectively.
